# Supplementary material for: Proteomic Determinants of Variation in Cholesterol Efflux: Observations from the Dallas Heart Study
Source: Int J Mol Sci. 2023 Oct 24;24(21):15526. doi: 10.3390/ijms242115526 (PMC10648649; doi:10.3390/ijms242115526)
Supplement: Supplementary file 1 [file ijms-24-15526-s001.zip › ijms-2639603-supplementary.pdf]

Supplementary data

Figure S1. CEC in individuals with persistent- extreme High and Low CEC.

a. CEC of apoB depleted plasma in individuals with extreme high and low CEC. **b.** Fractionated CEC (CEC- AUC (area under the curve))of SEC fractionated plasma using superose 6 FPLC column (35 fractions) in extreme high and low CEC groups. Red represent High CEC group and Blue represent Low CEC group. These results have been described previously in details (El-Ghazali, A.; Deodhar, S.; Saldanha, S.; Smyth, B.; Izbrand, M.; Gangwar, A.; Pahlavani, M.; Rohatgi, A. Molecular Patterns of Extreme and Persistent Cholesterol Efflux Capacity. Arterioscler Thromb Vasc Biol 2021, 41, 2588-2597, doi:10.1161/ATVBAHA.120.315648.).

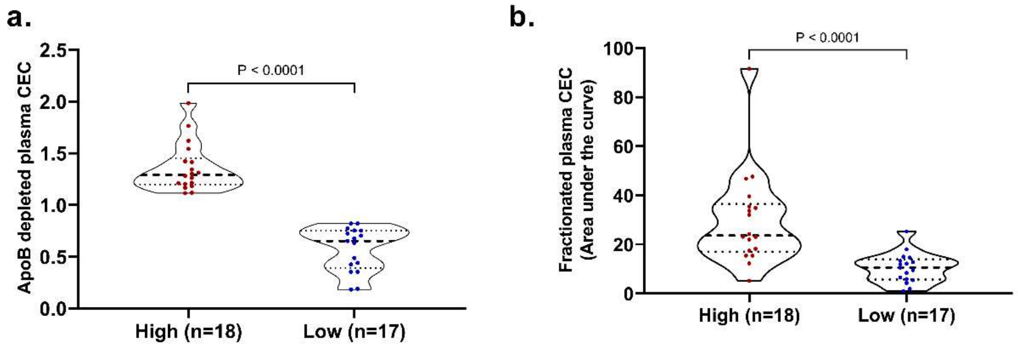

We observed a ~ two fold difference for apoB depleted plasma CEC (high: 1.29, low: 0.65) and > 2 fold difference for SEC fractionated plasma CEC-AUC (high: 23.64, low: 10.56) between extreme high and low CEC groups.

Figure S2. ApoA-I associated proteins abundances in individuals with Extreme high and Low CEC.

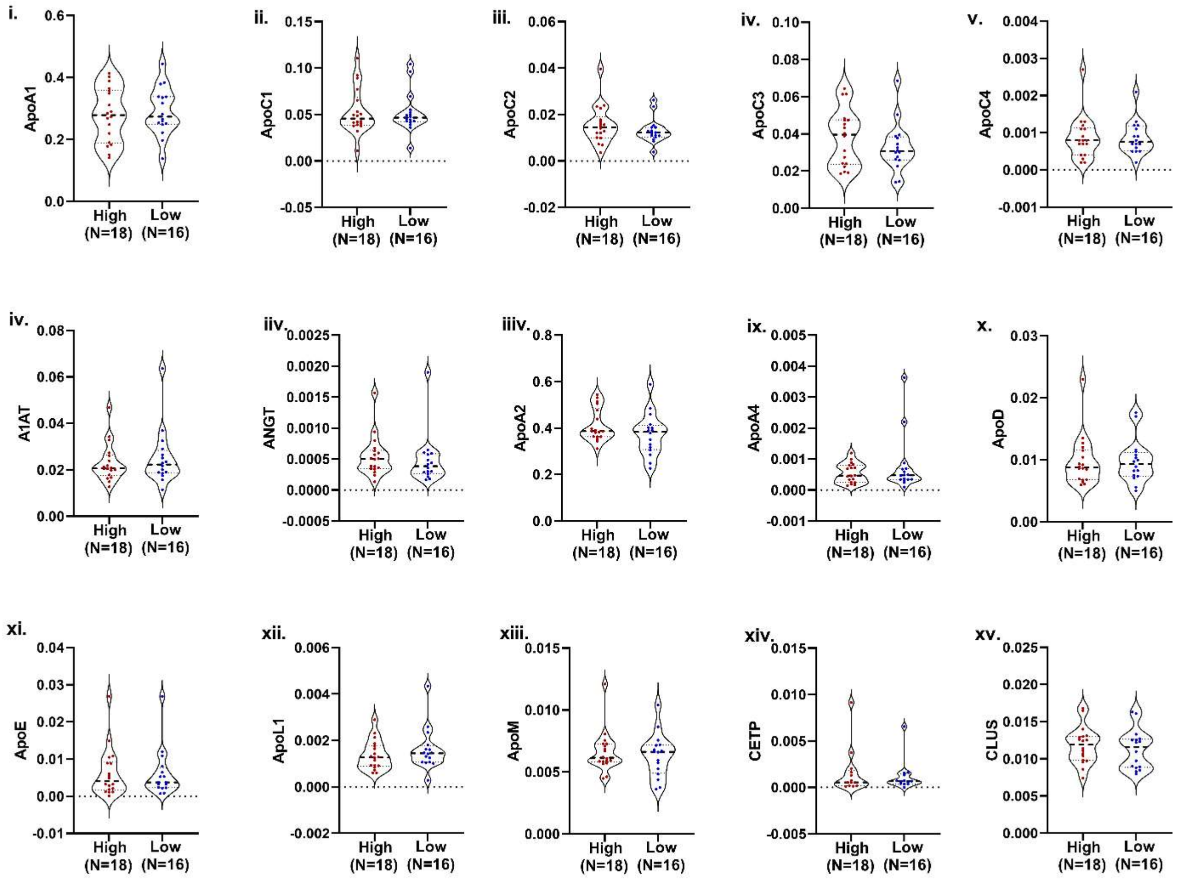

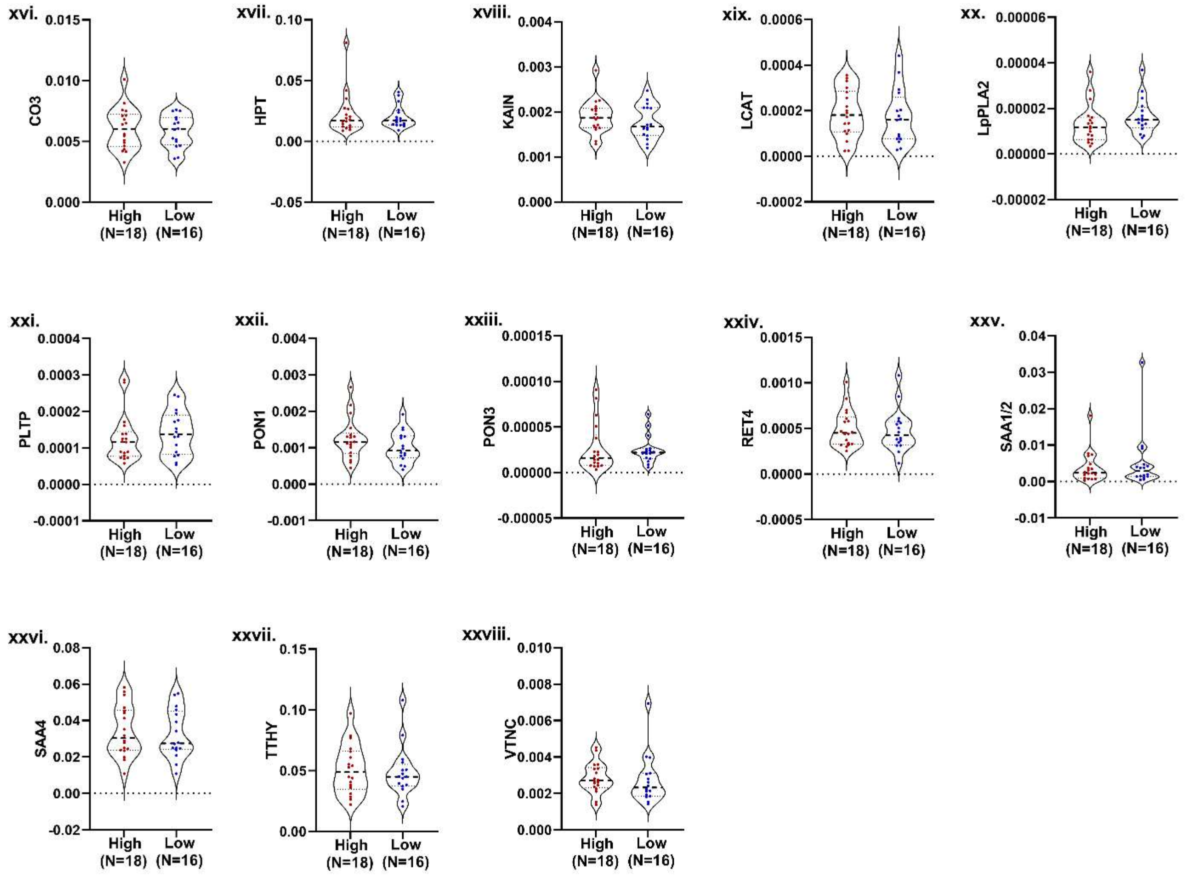

Figure S2. ApoA-I associated proteins abundances in individuals with extreme low and high CEC. Red represent High CEC group and blue represent low CEC group. The apoA-I sooociated proteins abundances were determined using method developed by Collier et al using His-tagged lipid free apoA-I dipping experiment followed by mass spectrometry (Collier, T.S.; Jin, Z.; Topbas, C.; Bystrom, C. Rapid Affinity Enrichment of Human Apolipoprotein A-I Associated Lipoproteins for Proteome Analysis. J Proteome Res 2018, 17, 1183-1193, doi:10.1021/acs.jproteome.7b00816.).

No significant difference was observed in abundances of apoA-I associated proteins between extreme high and low CEC groups.

Figure S3. AI-Lp subspecies in individuals with extreme high and low CEC.

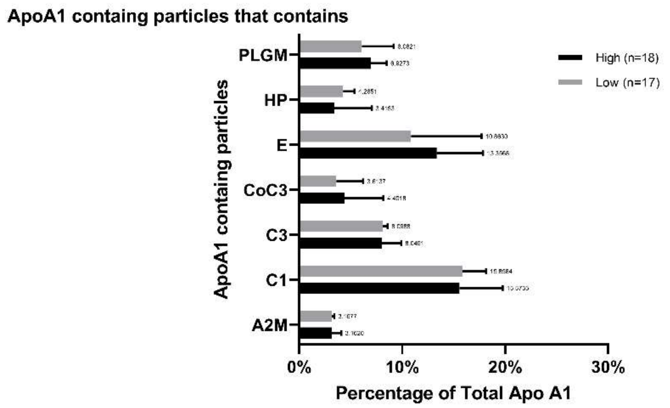

Figure S3: Bar graph showing AI-Lp subspecies as percentage of total ApoA1 present in individuals with extreme High and Low CEC. PLG: Plasminogen, HP: Haptoglobin, E: apoE, CO3: Complement C3, C3: apoC-III, C1: apoC-I, A2M: Alpha2Macroglubulin. Lp subspecies abundances were determined using Novel ELISA developed by Sacks et al (Sacks, F.M.; Liang, L.; Furtado, J.D.; Cai, T.; Davidson, W.S.; He, Z.; McClelland, R.L.; Rimm, E.B.; Jensen, M.K. Protein-Defined Subspecies of HDLs (High-Density Lipoproteins) and Differential Risk of Coronary Heart Disease in 4 Prospective Studies. Arterioscler Thromb Vasc Biol 2020, 40, 2714-2727, doi:10.1161/ATVBAHA.120.314609.).

AI- No significant difference was observed in abundances of seven AI-Lp subspecies between extreme low and high CEC groups.

Table S1: Correlation of apoA-I associated proteins, AI-Lp subspecies (apoA-I with and without certain proteins) with apo-B depleted plasma CEC (plasma CEC) in extreme high and low CEC groups.

	High Efflux		Low Efflux		Δ(high - low)
	Spearman r	p value	Spearman r	p value	Spearman r
**ApoC2**	0.49	0.04	-0.46	0.07	0.95
**ApoC3**	0.41	0.09	-0.41	0.11	0.82
**ApoC4**	0.17	0.49	-0.21	0.43	0.38
**ApoE**	0.27	0.28	-0.06	0.81	0.33
**ANGT**	0.02	0.94	-0.26	0.32	0.28
**ApoC1**	0.14	0.57	-0.09	0.74	0.23
**HPT**	0.11	0.65	-0.05	0.86	0.16
**CLUS**	-0.15	0.56	-0.27	0.32	0.12
**LCAT**	0.30	0.22	0.20	0.45	0.10
**ApoL1**	0.17	0.49	0.10	0.71	0.08
**TTHY**	0.31	0.21	0.24	0.36	0.07

**PON3**	-0.04	0.89	-0.05	0.87	0.01
**RET4**	-0.01	0.97	0.00	0.99	-0.01
**PON1**	0.15	0.54	0.18	0.51	-0.02
**KAIN**	0.10	0.70	0.16	0.55	-0.06
**CO3**	0.10	0.70	0.16	0.55	-0.06
**ApoA1**	-0.09	0.72	-0.02	0.94	-0.07
**A1AT**	0.13	0.60	0.21	0.42	-0.08
**ApoM**	0.15	0.56	0.29	0.27	-0.14
**SAA1/2**	-0.19	0.45	0.04	0.87	-0.23
**LpPLA2**	-0.10	0.70	0.26	0.33	-0.36
**SAA4**	0.08	0.74	0.47	0.07	-0.39
**ApoA2**	-0.13	0.60	0.36	0.17	-0.49
**ApoD**	-0.33	0.19	0.19	0.48	-0.51
**VTNC**	0.01	0.96	0.58	0.02	-0.57
**PLTP**	-0.40	0.10	0.22	0.42	-0.62
**ApoA4**	-0.21	0.39	0.66	0.01	-0.87
**WPA1**	-0.18	0.47	0.11	0.67	-
**A1_w_A2M**	0.05	0.84	0.14	0.59	-
**A1_w_ApoC1**	-0.07	0.78	0.37	0.14	-
**A1_w_ApoC3**	0.08	0.76	0.33	0.20	-
**A1_w_CO3**	0.02	0.94	0.28	0.27	-
**A1_w_ApoE**	-0.05	0.84	0.02	0.95	-
**A1_w_HP**	0.13	0.60	-0.08	0.75	-
**A1_w_PLG**	0.19	0.45	0.08	0.76	-
**A1_wo_A2M**	-0.20	0.42	0.15	0.56	-
**A1_wo_ApoC1**	-0.14	0.57	0.10	0.69	-
**A1_wo_ApoC3**	-0.23	0.36	0.16	0.55	-
**A1_wo_CO3**	-0.21	0.41	0.14	0.60	-
**A1_wo_ApoE**	-0.20	0.43	0.14	0.60	-
**A1_wo_HP**	-0.21	0.40	0.19	0.47	-
**A1_wo_PLG**	-0.20	0.42	0.18	0.48	-

Table S1: ApoA-I associated proteins and AI-Lp subspecies abundance correlation with Apo-B depleted plasma CEC (plasma CEC) in individuals with extreme high and low CEC. Table showing spearman correlation coefficient (spearman r). WPA: Total plasma ApoA-I (mg/dL), A1_w_A2M: ApoA-I (mg/dL) in HDL that contains alpha-2-macroglobulin, A1_w_ApoC-I: ApoA-I (mg/dL) in HDL that contains ApoC-I, A1_w_ApoC3: ApoA-I (mg/dL) in HDL that contains ApoC-III, A1_w_CO3: ApoA-I (mg/dL) in HDL that contains complement C3, A1_w_ApoE: ApoA-I (mg/dL) in HDL that contains ApoE, A1_w_HP: ApoA-I (mg/dL) in HDL that contains haptoglobin, A1_w_PLG: ApoA-I (mg/dL) in HDL that contains plasminogen, A1_wo_A2M: ApoA-I (mg/dL) in HDL that lacks alpha-2-macroglobulin, A1_wo_ApoC-I: ApoA-I (mg/dL) in HDL that lacks ApoC-I, A1_wo_ApoC3: ApoA-I (mg/dL) in HDL that lacks ApoC-III, A1_wo_CO3: ApoA-I (mg/dL) in HDL that lacks complement C3, A1_wo_ApoE: ApoA-I (mg/dL) in HDL that lacks ApoE,

A1_wo_HP: ApoA-I (mg/dL) in HDL that lacks haptoglobin, A1_wo_PLG: ApoA-I (mg/dL) in HDL that lacks plasminogen, Plasma CEC: ApoB depleted plasma cholesterol efflux capacity.

Table S2. Apo A-I associated proteins and AI-Lp subspecies (apoA-I with and without certain protein) correlation with fractionated CEC area under curve (CEC-AUC).

	High Efflux AUC		Low Efflux AUC		Δ (high - low)
	Spearman r	p value	Spearman r	p value	Spearman r
**CLUS**	0.32	0.20	-0.09	0.73	0.41
**SAA1/2**	0.30	0.23	-0.05	0.87	0.35
**PLTP**	0.16	0.52	-0.12	0.65	0.29
**ApoC2**	0.27	0.27	0.00	1.00	0.27
**ApoD**	0.22	0.37	-0.04	0.88	0.27
**ApoL1**	0.27	0.28	0.00	0.99	0.26
**HPT**	0.26	0.30	0.05	0.87	0.21
**ApoA4**	0.14	0.59	-0.07	0.80	0.21
**ApoC4**	0.21	0.40	0.03	0.91	0.18
**CO3**	0.20	0.43	0.02	0.94	0.18
**LpPLA2**	0.09	0.72	-0.05	0.86	0.14
**TTHY**	-0.04	0.87	-0.17	0.53	0.13
**PON1**	0.42	0.08	0.33	0.22	0.10
**ANGT**	-0.05	0.84	-0.11	0.67	0.06
**SAA4**	0.33	0.18	0.27	0.31	0.06
**ApoE**	0.21	0.40	0.16	0.55	0.05
**RET4**	0.18	0.46	0.15	0.57	0.03
**ApoA1**	0.17	0.49	0.17	0.53	0.01
**ApoM**	0.08	0.76	0.12	0.66	-0.04
**VTNC**	0.02	0.94	0.06	0.81	-0.05
**ApoC1**	0.12	0.63	0.24	0.36	-0.12
**ApoA2**	0.10	0.69	0.25	0.34	-0.15
**ApoC3**	-0.07	0.77	0.10	0.71	-0.17
**KAIN**	-0.13	0.60	0.08	0.78	-0.21
**LCAT**	0.03	0.89	0.26	0.34	-0.22
**A1AT**	0.04	0.87	0.36	0.18	-0.32
**PON3**	0.11	0.67	0.48	0.06	-0.37
**WPA1**	0.21	0.41	0.24	0.36	-
**A1_w_A2M**	0.24	0.33	0.00	0.99	-
**A1_w_ApoC1**	-0.32	0.20	0.16	0.54	-
**A1_w_ApoC3**	0.13	0.60	-0.06	0.81	-
**A1_w_CO3**	-0.15	0.55	-0.38	0.13	-
**A1_w_ApoE**	0.01	0.97	-0.28	0.28	-
**A1_w_HP**	0.42	0.08	0.03	0.90	-

**A1_w_PLG**	0.21	0.41	-0.19	0.45	-
**A1_wo_A2M**	0.22	0.38	0.28	0.28	-
**A1_wo_ApoC1**	0.25	0.32	0.38	0.13	-
**A1_wo_ApoC3**	0.21	0.41	0.33	0.20	-
**A1_wo_CO3**	0.19	0.45	0.45	0.07	-
**A1_wo_ApoE**	0.24	0.35	0.58	0.02	-
**A1_wo_HP**	0.18	0.47	0.33	0.20	-
**A1_wo_PLG**	0.20	0.44	0.50	0.04	-

Table S2: ApoA-I associated proteins and AI-Lp subspecies abundance correlation with fractionated CEC (CEC-AUC) in individuals with extreme high and low CEC. Table showing spearman correlation coefficient (spearman r). WPA: Total plasma ApoA-I (mg/dL), A1_w_A2M: ApoA-I (mg/dL) in HDL that contains alpha-2-macroglobulin, A1_w_ApoC-I: ApoA-I (mg/dL) in HDL that contains ApoC-I, A1_w_ApoC3: ApoA-I (mg/dL) in HDL that contains ApoC-III, A1_w_CO3: ApoA-I (mg/dL) in HDL that contains complement C3, A1_w_ApoE: ApoA-I (mg/dL) in HDL that contains ApoE, A1_w_HP: ApoA-I (mg/dL) in HDL that contains haptoglobin, A1_w_PLG: ApoA-I (mg/dL) in HDL that contains plasminogen, A1_wo_A2M: ApoA-I (mg/dL) in HDL that lacks alpha-2-macroglobulin, A1_wo_ApoC-I: ApoA-I (mg/dL) in HDL that lacks ApoC-I, A1_wo_ApoC-III: ApoA-I (mg/dL) in HDL that lacks ApoC-III, A1_wo_CO3: ApoA-I (mg/dL) in HDL that lacks complement C3, A1_wo_ApoE: ApoA-I (mg/dL) in HDL that lacks ApoE, A1_wo_HP: ApoA-I (mg/dL) in HDL that lacks haptoglobin, A1_wo_PLG: ApoA-I (mg/dL) in HDL that lacks plasminogen, Fractionated plasma CEC: Area under curve for total efflux of the FPLC fractions (30-69) collected from Apo B depleted plasma SEC using superpose 6 column.

Supplementary methods

Preparation of apoB depleted plasma

Polyethylene glycol (PEG) precipitation method was used to deplete EDTA plasma of apolipoprotein B (apoB) containing lipoproteins. Briefly, 20% polyethylene glycol (molecular weight 6000, Sigma-Aldrich) prepared in 200 mmol/L, pH 7.4 glycine buffer was added to whole plasma (plasma:polyethylene glycol, 10:4 ratio), incubated at room temperature for 20 minutes and centrifuged at 16000 rpm for 30 minutes at 4 °C. The supernatant was then transferred to a separate tube and centrifuged a second time for

complete removal of apoB-containing lipoproteins. Finally, the supernatant was collected and used for further experiments.

Separation of Lipoprotein using FPLC

Size exclusion chromatography was performed using Superose 6 increase column (10/300 GL; GE Healthcare) on an ÄKTA pure protein purification system (GE Healthcare) to separate lipoproteins. 500 μL of fresh apoB-depleted plasma was applied to a single Superose 6 column with a flow rate of 0.4 mL/min, detection wavelength 280 nm, and at 4 °C. Samples were eluted in filtration buffer containing 20 mmol/L NaH2PO4, 30 mmol/L Na2HPO4, 150 mmol/L NaCl. Fraction collector F9-C (GE Healthcare) was used to collect a total of 77 fractions (300 μL per fraction).

Measurement of BODIPY Cholesterol efflux capacity

CEC was measured using BODIPY cholesterol method as described previously[1]. Briefly, CEC was measured as efflux of BODIPY (Avanti polar lipids) from J774 murine macrophages (ATCC) to an appropriate acceptor. J774 macrophages were grown with RPMI supplemented with 10% FBS and were seeded into 96-well plates at a density of 70 000 cells/well. The cells were allowed to divide for an additional 24 hours until reaching 90% to 100% confluence under standard cell culture conditions (5% CO2, 25% humidity and at 37°C temperature). On the subsequent day, a mixture containing fluorescence-labeled cholesterol, BODIPY (Avanti polar lipids), 20% BSA, and ACAT inhibitor were added to the cells. After 1 hour, MEM-hepes buffer was used to wash the cells twice followed by a 24-h incubation in cAMP media. On the third day, MEM-hepes buffer was used to wash cells again. Then these cells were incubated at 37 °C with a cholesterol acceptor for 4 hours without CO2. The current study employed fresh ApoB-depleted

plasma and the individual fresh fast performance liquid chromatography (FPLC) fractions obtained after each sample were subjected to SEC as cholesterol acceptor. After incubating the samples for 4-h, fluorescence (Ex: 482 nm, Em: 515 nm) of efflux media was read using a microplate reader. The cultured monolayers were solubilized with NaOH and H2O. Fluorescence was measured to account for BODIPY-cholesterol remaining inside of the cells. At time zero, the total BODIPY-cholesterol incorporated into cells is the sum of cholesterol removed from cells (efflux) and that remaining inside of the cells. A set of controls (media containing no cholesterol acceptors) were included in each plate to account for BODIPY-cholesterol background fluorescence. To control for inter-assay variability, an additional apoB-depleted plasma sample from pooled healthy volunteers was used on each plate. Further, a recombinant human apoA-I protein (Sigma, SRP4693) was used at 7 increasing concentrations (1–100 µg/mL) as a cholesterol acceptor. This served as a standard curve on each plate. We subtracted background from efflux measurements of all acceptors and then divided the difference by the total BODIPY-cholesterol incorporated in each cell at time zero to calculate percent efflux. Efflux measurements were then normalized to efflux elicited by apoA-I concentration of 50 µg/mL. Previous bioinformatics analysis showed that this concentration, when used for normalization, yielded minimal signal to noise ratio. Only fractions No. 30 to 69 were utilized for the fraction-specific measurements, as previous studies showed that these were the only fractions that elicited efflux[1].

1. El-Ghazali, A.; Deodhar, S.; Saldanha, S.; Smyth, B.; Izbrand, M.; Gangwar, A.; Pahlavani, M.; Rohatgi, A. Molecular Patterns of Extreme and Persistent Cholesterol Efflux Capacity. *Arterioscler Thromb Vasc Biol* **2021**, *41*, 2588-2597, doi:10.1161/ATVBAHA.120.315648.
